# An evaluation of dexmedetomidine in combination with midazolam in pediatric sedation: a systematic review and meta-analysis

**DOI:** 10.1186/s12871-024-02570-1

**Published:** 2024-06-21

**Authors:** Juan Nie, Chenxi Li, Ge Yang, Huihui Chang, Guicong Ding

**Affiliations:** 1 Shenzhen Children’s Hospital, Shenzhen, 518026 China; 2 Department of Pediatric Dentistry, Sichuan Hospital of Stomatology, Chengdu, 61000 China; 3 Oncological Department of Oral and Maxillofacial Surgery, School of Stomatology, Stomatology Research Institute of Xinjiang Autonomous Region, The First Affiliated Hospital of Xinjiang Medical University, Xinjiang Medical University, Urumqi, 830054 People’s Republic of China

**Keywords:** Dexmedetomidine, Midazolam, Pediatrics, Sedation, Meta-analysis

## Abstract

**Background:**

Dexmedetomidine and midazolam are commonly used sedatives in children. We conducted a systematic review and meta-analysis to compare the safety and effectiveness of sedation provided by dexmedetomidine combined with midazolam versus other sedatives including chloral hydrate, midazolam and other sedatives in pediatric sedation.

**Methods:**

The Embase, Web of Science, Cochrane Library, and PubMed databases, and Clinicaltrials.gov register of controlled trials were searched from inception to June 2022. All randomized controlled trials used dexmedetomidine-midazolam in pediatric sedation were enrolled. The articles search, data extraction, and quality assessment of included studies were performed independently by two researchers. The success rate of sedation was considered as the primary outcome. The secondary outcomes included onset time of sedation, recovery time of sedation and occurrence of adverse events.

**Results:**

A total of 522 studies were screened and 6 RCTs were identified; 859 patients were analyzed. The administration of dexmedetomidine combined with midazolam was associated with a higher sedation success rate and a lower incidence of nausea and vomiting in computed tomography, magnetic resonance imaging, Auditory Brainstem Response test or fiberoptic bronchoscopy examinations than the other sedatives did (OR = 2.92; 95% CI: 1.39–6.13, *P* = 0.005, I^2^ = 51%; OR = 0.23, 95% CI: 0.07–0.68, *P* = 0.008, I^2^ = 0%, respectively). Two groups did not differ significantly in recovery time and the occurrence of adverse reactions (WMD = − 0.27, 95% CI: − 0.93 to − 0.39, *P* = 0.42; OR 0.70; 95% CI: 0.48–1.02, *P* = 0.06, I^2^ = 45%. respectively). However, the results of the subgroup analysis of ASA I-II children showed a quicker onset time in dexmedetomidine-midazolam group than the other sedatives (WMD=−3.08; 95% CI: −4.66 to − 1.49, *P* = 0.0001, I^2^ = 30%).

**Conclusions:**

This meta-analysis showed that compared with the control group, dexmedetomidine combined with midazolam group provided higher sedation success rates and caused a lower incidence of nausea and vomiting in completing examinations, indicating a prospective outpatient clinical application for procedural sedation.

## Introduction

Children constitute a unique patient group characterized by their young age, ongoing physical and mental development, and often, a significant anxiety about being separated from their parents. This combination generally results in a low tolerance and heightened sensitivity towards medical examinations and treatments, particularly when it comes to invasive procedures like dental treatments, laryngoscopies, and so forth. The anxiety and discomfort of children greatly increase the clinical risk, and reduce the efficiency and quality of clinical diagnosis and treatment. Procedural sedation or anesthesia have to be required for these uncooperative children. However, procedural sedation can be performed outside the operating room, and relatively low cost is an additional benefit [1]. As a result, the use of pharmacological sedation has increasingly garnered attention in clinical practice.

Many sedatives have been recommended for pediatric sedation, such as chloral hydrate, midazolam, propofol, dexmedetomidine, and so on [2]. Previously, oral chloral hydrate was commonly used in pediatric sedation. However, it often causes adverse reactions such as nausea, vomiting, and stomachache in children because of its stimulation on upper gastrointestinal mucosa [3]. Therefore, its application in children is greatly limited. Midazolam is one of commonly used benzodiazepine sedatives, with sedative, hypnotic and anti-anxiety [4]. It has the advantages of rapid onset, high metabolic clearance and anterograde amnesia, and it is commonly used in pediatric sedation [5]. However, adverse effects including cognitive impairment, nausea, vomiting, respiratory depression, and postoperative emergence agitation have been reported in children after using midazolam [6]. Propofol is a widely-used sedative agent in pediatric sedation for various medical and diagnostic procedures [7]. Its application in children is valued for its rapid onset and short duration of action, allowing for quick recovery post-procedure [8]. However, its use is limited by the need for vigilant monitoring of respiratory and cardiovascular effects and requires expertise in administration [7, 8]. Dexmedetomidine (DEX) is a new type of sedative drug and a highly specific and selective α2-adrenergic receptor agonist with sedative and mild analgesic properties [9]. The sedation produced by DEX is in the locus coeruleus and similar to natural sleep [10], indicating it has little respiratory depression and hemodynamics effects. Therefore, DEX can be used as an auxiliary drug for opioids or benzodiazepines [11, 12].

Although several studies have reported the sedative effect of the combination of DEX and midazolam in children, but in many reports, the included amount patients were small, and the conclusions were controversial [13, 14]. Nonetheless, no meta-analysis has been performed to assess the outcomes of these studies. In our study, we have conducted a meta-analysis to thoroughly evaluate the safety and efficacy of combining DEX with midazolam for procedural sedation in children. This is aimed at offering an evidence-based reference for the clinical, rational use of these drugs.

## Materials and methods

This meta-analysis was conducted following in the Preferred Reporting Items for Systematic Reviews and Meta-Analyses (PRISMA) and the Cochrane Review Methods.

### Inclusion criteria

Study selection followed these criteria: (1) Participants: the patients were the children under 18 years old (regardless of the different surgical or diagnostic procedures) (2). Intervention and comparison: DEX in combination with midazolam (regardless of the route and dose of administration) in an intervention group; other sedatives such as midazolam, chloral hydrate, propofol, or pentobarbital administered in control group (3). Outcome measures: the primary outcome was the success rate of sedation, depended on factors such as the depth of sedation, procedural requirements, patient satisfaction, and safety. The secondary outcomes were as follows: (a) onset time of sedation, (b) recovery time of sedation, (c) occurrence of adverse events, including bradycardia, hypotension, nausea and vomiting (4). Study design: prospective randomized controlled trials with no language limitations.

### Data sources and literature sources

Two investigators have independently searched the following databases (inception to 30 June 2022): Embase, Web of Science, Cochrane Library, and PubMed. There were no restrictions of language. The electronic search strategy was performed using the following keywords: pediatric, DEX, midazolam and randomized controlled trial. Detailed retrieval process was provided in Table 1. We also have checked the reference lists of the screened full-text studies to identify other potentially eligible trials. If some important information were not provided in the original literature, we would seek it from the corresponding authors through email.

**Table 1:** The detailed retrieval process

Search	Query	Items found
**Pubmed**		
#1	Infant [mesh]	1,217,850
#2	newborn* [tiab] or neonat* [tiab] or infant* [tiab] or infant [tiab] or baby [tiab] or babies [tiab] or toddler* [tiab]	820,275
#3	#1 OR #2	1,535,909
#4	Child [mesh]	2,073,723
#5	Children[tiab]	1,181,026
#6	#4 OR #5	2,397,032
#7	Pediatrics [mesh]	62,386
#8	p? ediatric* [tiab] or child* [tiab] or kindergar* [tiab] or preschool* [tiab] or id [tiab] or aids [tiab] or schoolchild* [tiab] or “school age” [tiab] or school age [tiab] or preteen* [tiab] or youth* [tiab] or prepubescent* [tiab]	1,829,119
#9	#6 OR #7 OR #8	2,843,446
#10	Adolescent [mesh]	2,174,414
#11	adolesc* [tiab] or teen* [tiab] or youth* [tiab] or underage* [tiab] or “under age*” [tiab] or minor* [tiab] or juvenile* [tiab] or pubert* [tiab] or pubescen* [tiab] or “young people*” [tiab] or “young person*” [tiab] or “young adult*” [tiab]	950,558
#12	#10 OR #11	2,725,610
#13	#3 OR #9 OR #12	5,060,511
#14	(Midazolam[tiab]) OR (Midazolam Maleate[tiab]) OR (Maleate, Midazolam[tiab]) OR (Dormicum[tiab]) OR (Versed[tiab])OR (Midazolam Hydrochloride[tiab]) OR (Hydrochloride, Midazolam[tiab]) OR (Ro 21-3981[tiab]) OR (Ro 21 3981[tiab]) OR (Ro 213,981[tiab])	15,211
#15	(Dexmedetomidine [tiab]) OR (MPV-1440[tiab]) OR (MPV 1440[tiab]) OR (MPV1440 [tiab]) OR (precedes[tiab]) OR (Dexmedetomidine Hydrochloride[tiab]) OR (Hydrochloride, Dexmedetomidine[tiab])	25,518
#16	(randomized controlled trial [pt] OR controlled clinical trial [pt] OR randomized [tiab] OR placebo [tiab] OR clinical trials as topic [mesh: noexp] OR randomly [tiab] OR trial [ti]) NOT (animals [mh] NOT humans [mh])	1,420,999
#17	#13AND #14 AND #135AND #16	138
**Embase**		
**Search**	**Query**	**Items found**
#1	‘Infant’/exp	1,227,543
#2	(newborn* or neonat* or infant* or infancy or baby or babies or toddler*):ab, ti	1,036,069
#3	#1 OR #2	1,641,603
#4	‘child’/exp	3,172,716
#5	**‘**children’:ti, ab	1,558,769
#6	#4 OR #5	3,501,284
#7	‘pediatrics’/exp	129,147
#8	(paediatric*or pediatric* or child* or kindergar* or preschool* or kid or kids or schoolchild* or ‘school age’ or schoolage or preteen* or youth* or prepubescent*):ab, ti	2,115,114
#9	#8 OR #7	2,181,515
#10	‘adolescent’/exp	1,821,037
#11	(adolesc* or teen* or youth* or underage* or “under age*” or minor* or juvenile* or pubert* or pubescen* or “young people*” or “young person*” or “young adult*”):ab, ti	1,207,744
#12	#10 OR #11	2,583,225
#13	#3 AND #6 AND #9 AND #12 AND [embase]/lim	86,881
#14	(dexmedetomidine: ab, ti OR ’mpv 1440’:ab, ti OR ’mpv1440’:ab, ti OR precedex: ab, ti OR ’dexmedetomidine hydrochloride’:ab, ti OR ’hydrochloride, dexmedetomidine’:ab, ti) AND [embase]/lim	9,580
#15	(midazolam: ab, ti OR dormicum: ab, ti OR ’midazolam maleate’:ab, ti OR ’maleate, midazolam’:ab, ti OR versed: ab, ti OR ’midazolam hydrochloride’:ab, ti OR ’hydrochloride, midazolam’:ab, ti OR ’ro 21-3981’:ab, ti OR ’ro 21 3981’:ab, ti OR ’ro 213,981’:ab, ti) AND [embase]/lim	21,106
#16	(‘randomized controlled trial’:ab, ti OR ’controlled clinical trial’:ab, ti OR ’randomized’:ab, ti OR placebo: ab, ti OR ’drug therapy’:ab, ti OR ’randomly’:ab, ti OR ’trial’:ab, ti OR ’groups’:ab, ti) AND [embase]/lim	3,909,282
#17	#13 AND #14 AND #15 AND #16	4
**Cochrane Library**		
**Search**	**Query**	**Items found**
#1	MeSH descriptor: [Infant] explode all trees	34,717
#2	(newborn* or neonat* or infant* or infancy or baby or babies or toddler*):ti, ab, kw	84,221
#3	#1 or #2	84,221
#4	MeSH descriptor: [Child] explode all trees	61,040
#5	(Children): ti, ab, kw	160,794
#6	#4 or #5	160,794
#7	MeSH descriptor: [Pediatrics] explode all trees	724
#8	(paediatric*or pediatric* or child* or kindergar* or preschool* or kid or kids or schoolchild* or ‘school age’ or schoolage or preteen* or youth* or prepubescent*):ti, ab, kw	180,917
#9	#7 or #8	181,105
#10	MeSH descriptor: [Adolescent] explode all trees	110,030
#11	(adolesc* or teen* or youth* or underage* or “under age*” or minor* or juvenile* or pubert* or pubescen* or “young people*” or “young person*” or “young adult*”):ti, ab, kw	223,313
#12	#10 or #11	223,313
#13	#3 or #6 or #9 or #12	382,276
#14	(Midazolam): ti, ab, kw OR (Midazolam Maleate): ti, ab, kw OR (Dormicum): ti, ab, kw OR (“versed”):ti, ab, kw OR (Midazolam Hydrochloride): ti, ab, kw	9273
#15	(Dexmedetomidine): ti, ab, kw OR (MPV-1440):ti, ab, kw OR (Precedex): ti, ab, kw OR (Dexmedetomidine Hydrochloride)	6287
#16	#13 and #14 and #15	300 (4 reviews; 296 Trials)
**Web Of Science**		
**Search**	**Query**	**Items found**
#1	TS=(Infant OR child OR pediatrics OR adolescent )	2,545,283
#2	TS=(Midazolam OR Dormicum ORMidazolam Maleate OR Maleate, Midazolam OR Versed OR Midazolam Hydrochloride OR Hydrochloride, Midazolam OR Ro 21-3981 OR Ro 21 3981 OR Ro 213,981)	37,177
#3	TS=(Dexmedetomidine OR MPV-1440 OR MPV 1440 OR MPV1440 OR Precedex OR Dexmedetomidine Hydrochloride OR Hydrochloride, Dexmedetomidine)	10,172
#4	TS=(randomized controlled trial OR controlled clinical trial OR randomized OR placebo OR drug therapy OR randomly OR trial OR groups)	7,822,344
#5	#1 AND #2 AND #3 AND #4	314

www.clinicaltrials.gov

8 Studies found for: Dexmedetomidine | Completed Studies | Studies With Results | Interventional Studies | Midazolam

### Data extraction

Two reviewers conducted the literature by titles and abstracts individually, and full manuscripts were evaluated carefully assessed to finalize eligibility. And then they extracted data from eligible papers independently and cross-checked with each other. Irrelevant records were excluded by the two reviewers after reviewing titles and abstracts. If there were disagreements on data abstraction and quality assessment between them, another third reviewer would resolve the differences. After the full texts of the remaining studies were obtained and perused, the relevant articles were identified. Only the values of the present defined primary and secondary outcomes, presented either as means and standard deviation or as counts of events were used in this study. If we could not retrieve the exact information in the studies, we would seek it from the corresponding author through email.

### Risk of bias assessment

Two reviewers independently evaluated the risk of bias. In accordance with the Cochrane risk-of-bias tool for randomized controlled trials, we evaluated the methodological quality of relevant studies, which includes the following aspects: random sequence generation, allocation concealment, blinding of participants and personnel, blinding of outcome assessment, incomplete outcome data, and selective reporting.

### Statistical analysis

Statistical analyses were performed using Review Manager 5.4 software from The Cochrane Collaboration. Dichotomous data were analyzed by odds ratio (OR) and 95% confidence interval (CI). Weighted mean differences (WMD) of mean values and standard deviations were calculated for Continuous data. A P value < 0.05 was considered statistically significant. For the impact of heterogeneity, the I-squared (I^2^) test was chosen for the estimation. If there was significant heterogeneity (I^2^ less than 50%), the fixed-effects model was applied; otherwise, the random-effects model was selected, and the sensitivity analysis was performed. We pre-specified several subgroup analyses to explore potential sources of heterogeneity: ASA physical status classification, and the occurrence of adverse reactions during sedation and post-procedure. Additionally, some post hoc subgroup analyses were performed, including the occurrence of bradycardia (yes vs. no) and hypotension (yes vs. no) during and after sedation. And we assessed the potential for publication bias through visual analysis of funnel plots. To account for Type I and Type II errors, as well as to reach a predetermined number of patients based on previous studies [15], we conducted a Trial Sequential Analysis (TSA). The TSA was configured with a power level of 90% and a two-sided alpha level of 0.05.

## Results

**Fig. 1:**
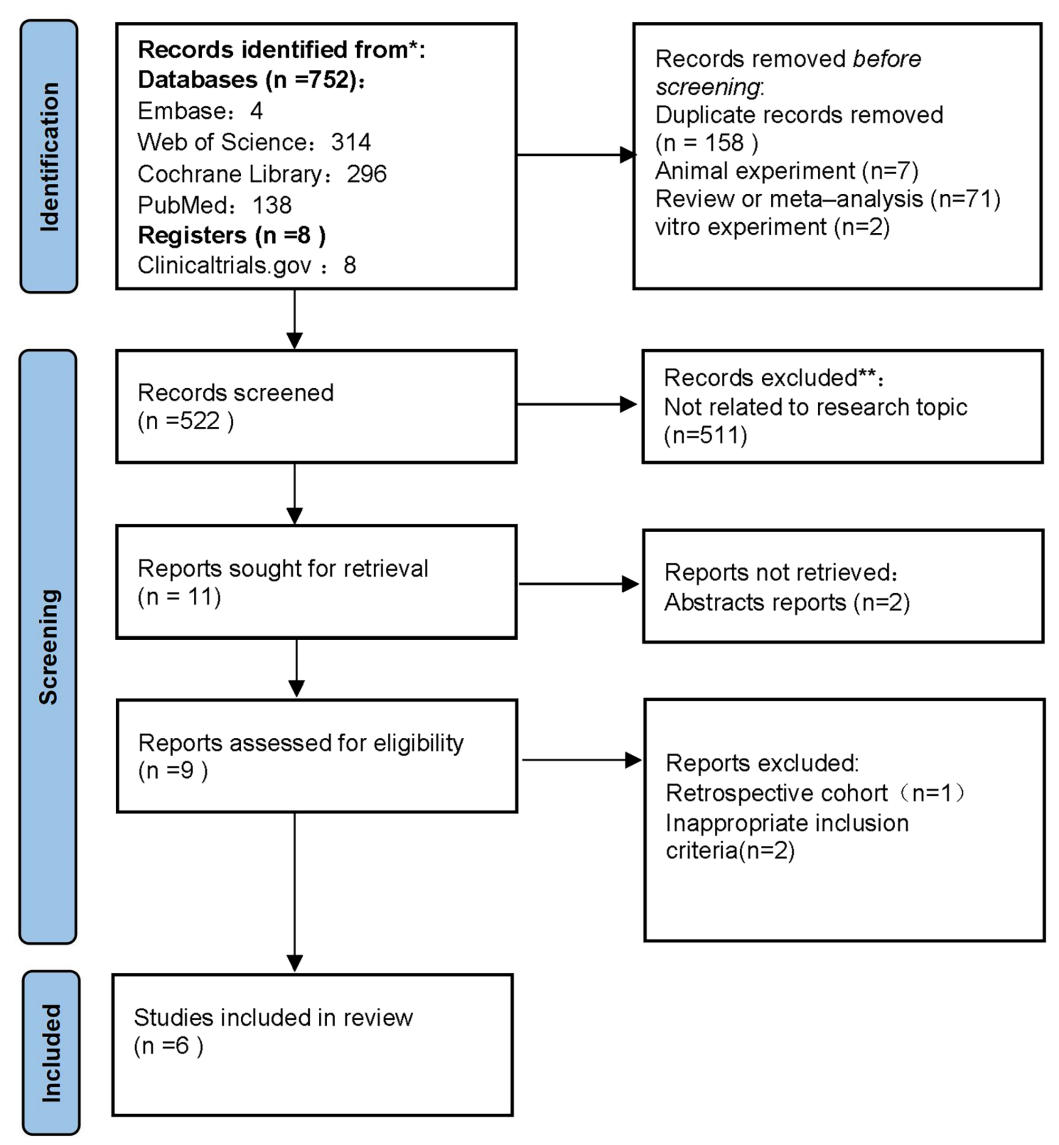
Flow diagram of the literature search strategy

As described in Fig. 1, a total of 760 studies were identified initially after the online searching by title, keywords, or abstract. One hundred fifty-eight duplicate records, 7 animal researches, 71 review or meta–analysis and 2 vitro experiment were removed. And then 513 items were excluded after reviewing the title and abstract. A number of 9 items were retrieved in full-text. Three trials were excluded by full-text reviewing, one of them was a retrospective cohort study and two of them reported inappropriate inclusion criteria. Finally, 6 studies were found eligible for the consequent analysis (Fig. 1).

### Study characteristics

The main characteristics of these included studies were summarized in Table 2. These randomized controlled trials were published from 2008 to 2021, recruited a total of 859 patients (ages ranged from 1 months to 12 years) were recruited. Among these, 254(29.6%) were girls and 605 (70.4%) were boys. 428 children received DEX-midazolam and 431 patients received the other sedatives, including 82 with chloral hydrate, 136 with DEX, 20 with midazolam-propofol, 93 with dexmedetomidine-(chloral hydrate), 60 with dexmedetomidine-propofol, and 40 by midazolam. The dosages and routes of sedatives were varied, as presented in Table 2. DEX was administered intranasally (1–3 µg/kg) or intravenously (0.5–0.7 µg/(kg·h)). Midazolam was delivered orally (0.3 mg/kg), intravenously (0.05-0.1 mg/kg), intranasally(0.3 mg/kg) or buccally (0.1-0.2 mg/kg). Chloral hydrate 50 mg/kg was orally administered. Propofol was delivered intravenously (67–300 µg/(kg·min)). The procedural sedation was assisted to complete the computed tomography (CT), magnetic resonance imaging (MRI), Auditory Brainstem Response (ABR) test, and fiberoptic bronchoscopy.

**Table 2:** Basic characteristics of 6 included studies

			Patients			intervention	Control	Outcome		
source	study design	Type of surgery/procedure	patient age(range)&ASA status	patients enrolled(gender: F/M, *n*)	Scales used for sedation measurement	DEX + MIDA group, dose, route of administration	other sedatives group, dose, route of administration	cases of adverse reactions	success rate	Funding and conflict of interest statement
Heard C 2008	RCT	MRI	aged 12–120 m (ASA I-II)	DEX + MIDA group(7/13,20); DEX + Propofol group(8/12,20)	Aldrete score	initial loading DEX (1ug/kg) followed by a continuous infusion DEX (0.5ug/(kg·h)) + IV MIDA(0.1 mg/kg)	initial loading DEX (1.0 ug/kg) followed by a continuous infusion DEX (0.5ug/(kg·h)) + IV propofol (250-300ug/(kg·min))	1/20 VS. 0/20	20/20 VS. 20/20	Funding: Departmental Funds. No conflict of interest was reported.
Li BL 2018	RCT (ChiCTR-TRC‐14,005,131)	ABR	aged 2–72 m (ASA I-II)	DEX + MIDA group(26/52,78); CH group(23/59,82)	UMSS; a 4-point behavioral scale; the Narcotrend	intranasal DEX(3ug/kg) + buccal MIDA(0.1 mg/kg)	oral CH (50 mg/kg) + intranasal 0.9% sodium chloride	15/78 VS. 17/82	70/78 VS. 57/82	No funding or conflict of interest was reported.
Li BL 2019	RCT (ChiCTR-TRC-14,004,761)	CT and/or ABR	aged 1–144 m (ASA I-II)	DEX + MIDA group(9/130,139); DEX group(14/122,136)	UMSS; a point behavioural scale; a four-point movement score	intranasal DEX(3ug/kg) + buccal MIDA(0.2 mg/kg)	intranasal DEX(3ug/kg) + buccal placebo	10/139 VS. 5/136	116/139 VS. 89/136	Funding: Guangzhou Health and Family Plan ning Commission Program, Guangzhou Women and Children’s Medical Center/Guangzhou Institute of Pediatrics. No conflict of interest was reported.
Ji YY, 2020	RCT	MRI	aged 36–96 m (ASA I-III)	DEX + MIDA group(41/52,93); DEX + CH group(29/64,93)	the Ramsay score	intranasal DEX(2ug/kg) + oral MIDA(0.3 mg/kg)	Oral CH (50 mg/kg) + intranasal DEX(2ug/kg)	8/93 VS. 5/93	88/93 VS. 92/93	Funding: Scientific Research Project of Shanghai Science and Technology Commission.No conflict of interest was reported.
Wu ZF 2020	RCT	MRI	aged 1–96 m (ASA I-II)	DEX + MIDA group(26/14,40); MIDA group(21/19,40)	the Ramsay score	intranasal DEX(3ug/kg) + intranasal MIDA(0.3 mg/kg)	intranasal MIDA(0.3 mg/kg)	5/40 VS. 7/40	38/40 VS. 30/40	Funding: the Clinical Research Foundation and Humani ties & Social Science Foundation of the Military Medical University. No conflict of interest was reported.
Zhang J 2021	RCT	fiberoptic bronchoscopy	aged 12–36 m (ASA I-II)	DEX + MIDA group(24/34,58); M + Propofol group(26/34,40)	the Berggren score; agitation score	intravenous pumping DEX(1ug/kg) and then intravenous injection MIDA(0.05 mg/kg) ,10 min later followed DEX(0.5-0.7ug/(kg·h))	intravenous pumping propofol (2 mg/kg) and then injection MIDA(0.05 mg/kg) ,10 min later followed propofol (at 4–6 mg/(kg·h))	23/58 VS. 31/60	56/58 VS. 50/60	No funding or conflict of interest was reported.

RCT, randomized controlled trials; ABR, auditory brainstem response; CT, computerized tomography; MRI, magnetic resonance imaging examination; DXM, dexmedetomidine; MIDA, midazolam; UMSS, University of Michigan Sedation Score

### Risk of bias assessment

**Fig. 2:**
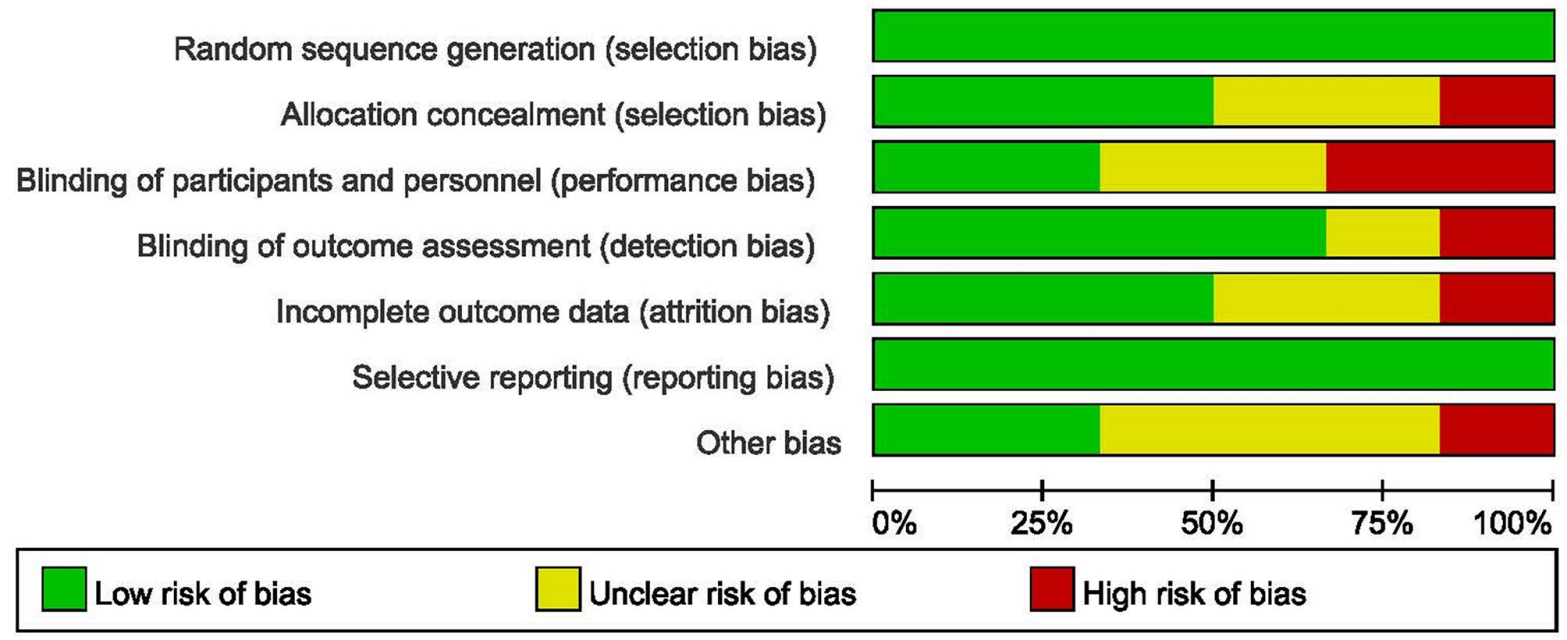
Summary risk assessment of literature bias

We evaluated included studies [14, 16–20] according to the Cochrane risk-of-bias tool to assess risk of bias, including random sequence generation, allocation concealment, blinding of participants and personnel, blinding of outcome assessment, attrition bias, reporting bias, and other bias. All studies reported the method of random sequence generation, and three of them described an adequate allocation concealment scheme in detail. Four trials mentioned the blinding procedure of participants and personnel and the blinding procedure of outcome assessment. Three of them were high-quality trials with low risk of bias in all items. One study was moderate-quality study and 2 studies were accessed as being low quality. The overall quality of included studies was moderate. The more detail of quality assessment was shown in Fig. 2.

### Primary outcomes

#### Success rate of sedation

**Fig. 3:**
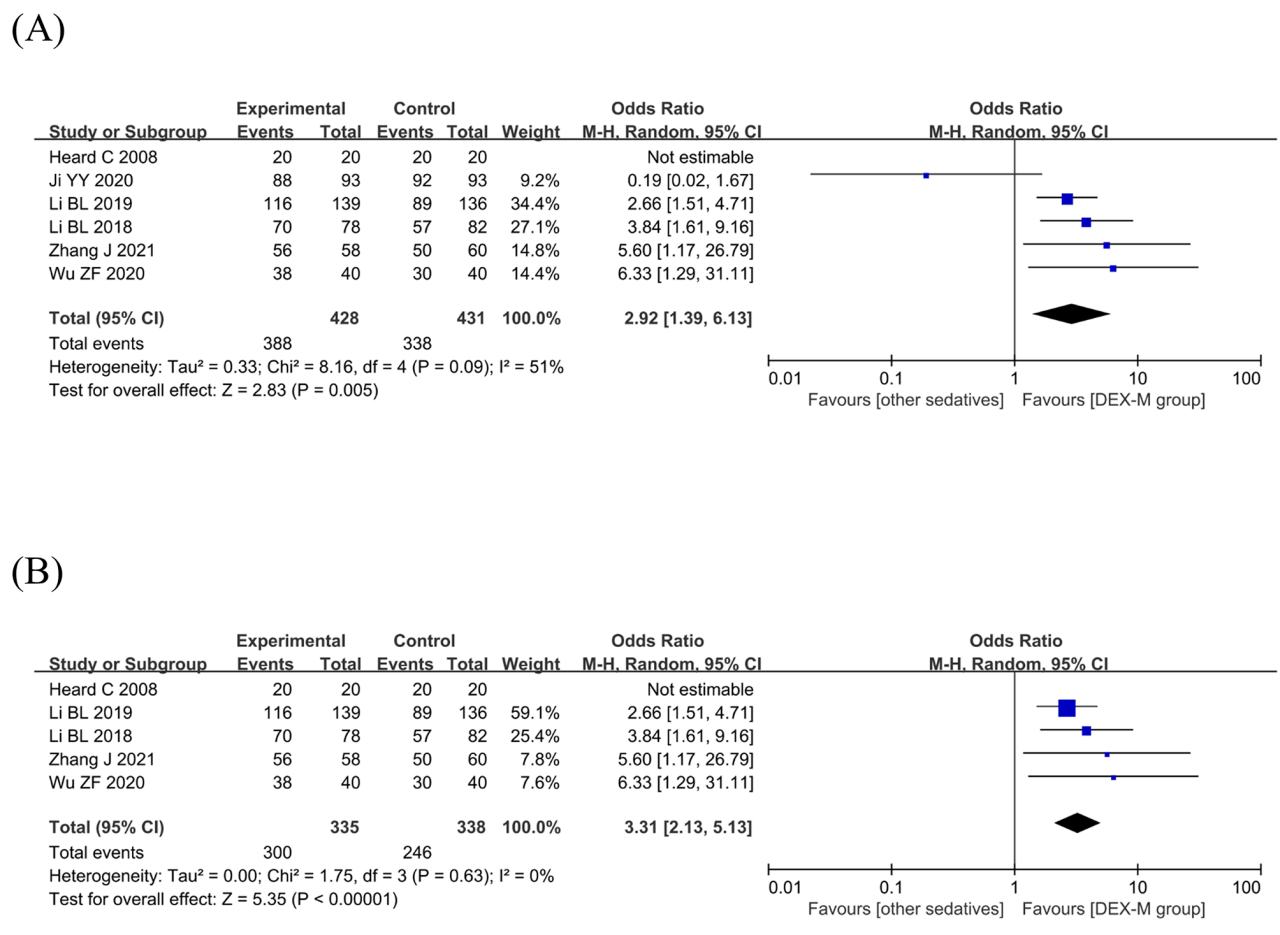
Efficacy of dexmedetomidine-midazolam vs. other sedatives sedation in children. (**A**) Success rate of sedation; (**B**) the sensitivity analysis of success rate of sedation

All six studies with 859 patients were analyzed about the sedation success rate. The I^2^ was equal to 51% (higher than 50%), demonstrating that statistical heterogeneity was existed among the studies. The random effects model was chosen for meta-analysis. The results of success rate of sedation in these included RCTs showed that sedation with dexmedetomidine-midazolam group by intranasal, intravenous, oral, or buccal routes had a statistically higher success rates than other sedatives groups (OR 2.92; 95% CI: 1.39–6.13, *P* = 0.005, I^2^ = 51%; Fig. 3A). In the sensitivity analysis, we excluded study of Ji YY 2020[15] and found that the value of I^2^ decreased to 0%, indicating that this trial had highly heterogeneity. After analyzing the full text carefully, it was found that the heterogeneity was mainly derived from clinical heterogeneity. The children included in the study of Ji YY 2020[15] were ASA physical status I to III, while other included studies were ASA grades I or II. Subgroup analysis still showed that the using of dexmedetomidine-midazolam was associated with higher success rate of sedation compared to other sedatives(OR 3.31; 95% CI: 2.13–5.13, *P*<0.00001, I^2^ = 0%; Fig. 3B).

### Secondary outcomes

#### Onset time of sedation

**Fig. 4:**
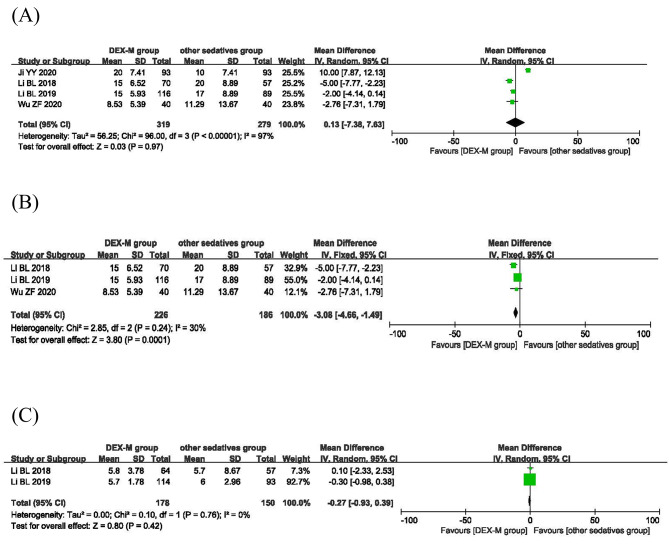
Efficacy of dexmedetomidine-midazolam vs. other sedatives sedation in children. (**A**) Onset time of sedation; (**B**) the subgroup analysis of onset time of sedation; (**C**) the recovery time of sedation

The onset time of sedation was reported in four studies. There was statistical heterogeneity among the studies (*P*<0.00001, I^2^ = 97%; Fig. 4A), so the random effect model was used for meta-analysis (Fig. 4A). The results showed that there was no statistically significant difference in the onset time of sedation in ASA I-III children given dexmedetomidine-midazolam compared with those receiving other sedatives. Subgroup analysis showed that there was no statistical heterogeneity in ASA I-II children among the studies (*P* = 0.24, I^2^ = 30%; Fig. 4B), so the fixed effects model was used for meta-analysis. Dexmedetomidine-midazolam showed significantly quicker onset time than the other sedatives in subgroup analysis (WMD=−3.08; 95% CI: −4.66 to − 1.49, *P* = 0.0001, I^2^ = 30%;Fig. 4B).

#### Recovery time of sedation

Two studies reported the recovery time of sedation. Due to no statistical heterogeneity between them (*P* = 0.76, I^2^ = 0%; Fig. 4C), the fixed effects model was adopted for meta-analysis. The result revealed that the difference of recovery time of sedation between dexmedetomidine-midazolam group and other sedatives group was not significant (WMD = − 0.27, 95% CI: − 0.93 to − 0.39, *P* = 0.42; Fig. 4C).

#### Occurrence of adverse events

**Fig. 5:**
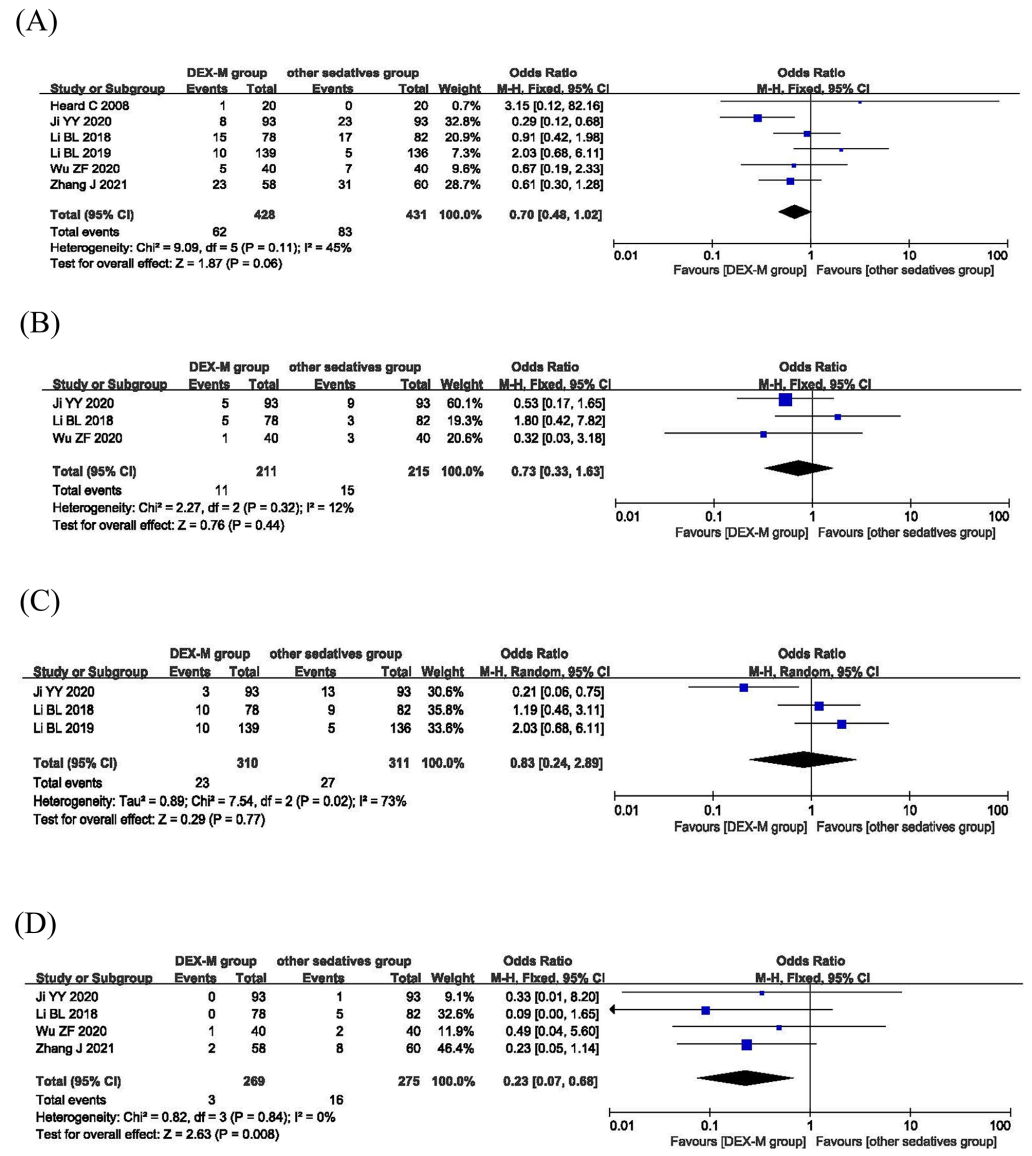
Safety of dexmedetomidine-midazolam vs. other sedatives sedation in children. (**A**) The occurrence of adverse reactions; (**B**) incidence of bradycardia; (**C**) incidence of hypotension; (**D**) incidence of nausea and vomiting

Adverse reactions were reported in all included trials. The fixed effects model was utilized for meta-analysis because of no statistical heterogeneity among the studies (*P* = 0.11, I^2^ = 45%; Fig. 5A). The results indicated that there was no significant statistical difference in the occurrence of adverse reactions between dexmedetomidine-midazolam sedation and other sedatives (OR 0.70; 95% CI: 0.48–1.02, *P* = 0.06, I^2^ = 45%; Fig. 5A). Subgroup analysis showed there was no significant statistical difference in the incidence of bradycardia between dexmedetomidine-midazolam group and control group (OR = 0.73; 95% CI: 0.33–1.63, *P* = 0.44, I^2^ = 12%; Fig. 5B). Three studies reported incidence of hypotension. As shown in Fig. 5C, the incidence of hypotension in dexmedetomidine-midazolam group was similar to other sedatives group, and the difference was not statistically significant (OR = 0.83; 95% CI: 0.24–2.89, *P* = 0.77, I^2^ = 73%; Fig. 5C). In the sensitivity analysis, we excluded study of Ji YY 2020[15] with high heterogeneity and found that the value of I^2^ decreased to 0%. However, there was still no remained significant difference in the incidence of hypotension between two groups (OR = 1.52; 95% CI: 0.74–3.14, *P* = 0.25, I^2^ = 0%). Five trials reported the occurrence of nausea and vomiting. A fixed effects model was chosen for meta-analysis for no statistical heterogeneity among the studies (*P* = 0.84, I^2^ = 0%; Fig. 5D). According to the results, DEX combined with midazolam showed a lower incidence rate of nausea and vomiting than other sedatives (OR = 0.23, 95% CI: 0.07–0.68, *P* = 0.008; Fig. 5D).

### Publication bias and trial sequential analysis

**Fig. 6:**
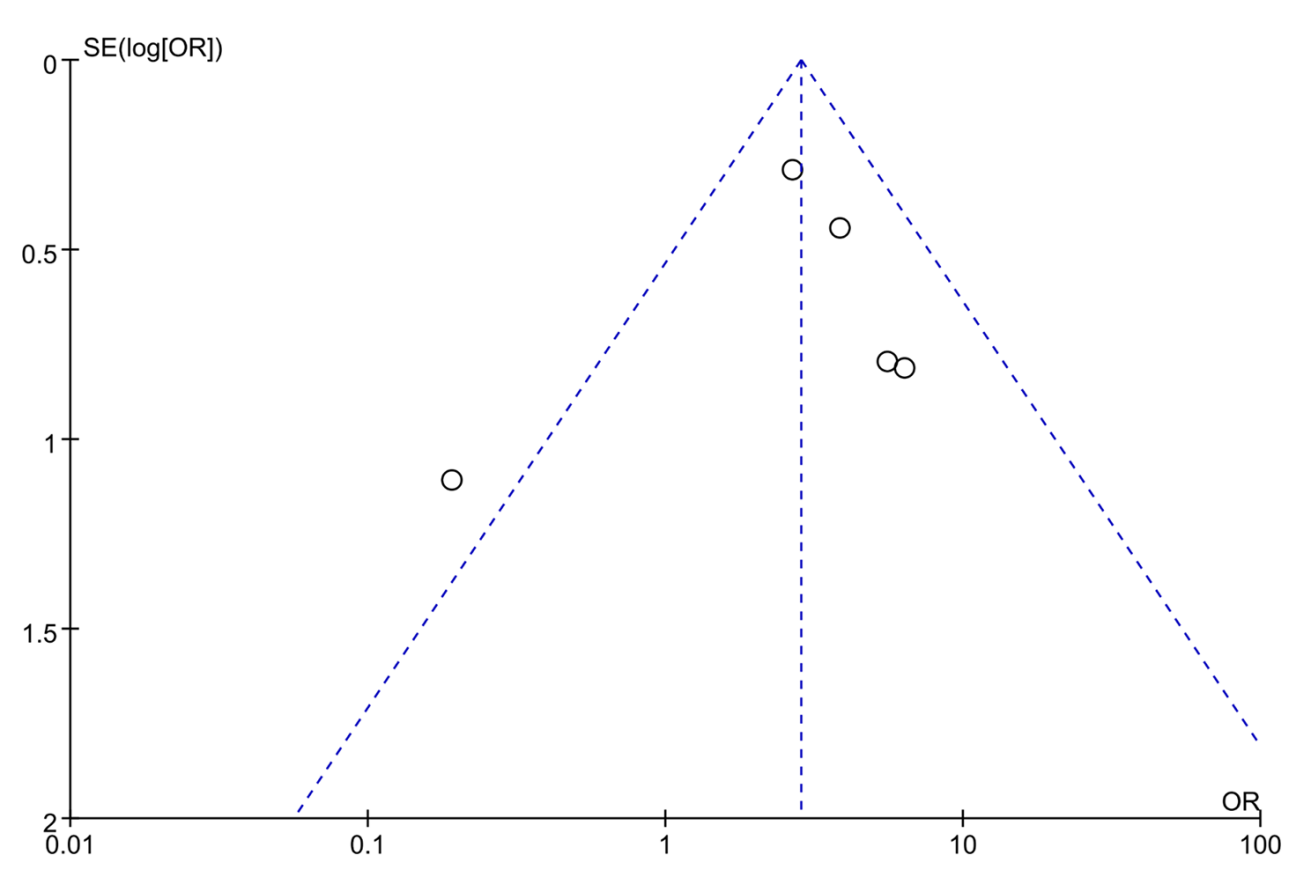
Funnel plot for the longitudinal observational studies on the success rate of sedation

**Fig. 7:**
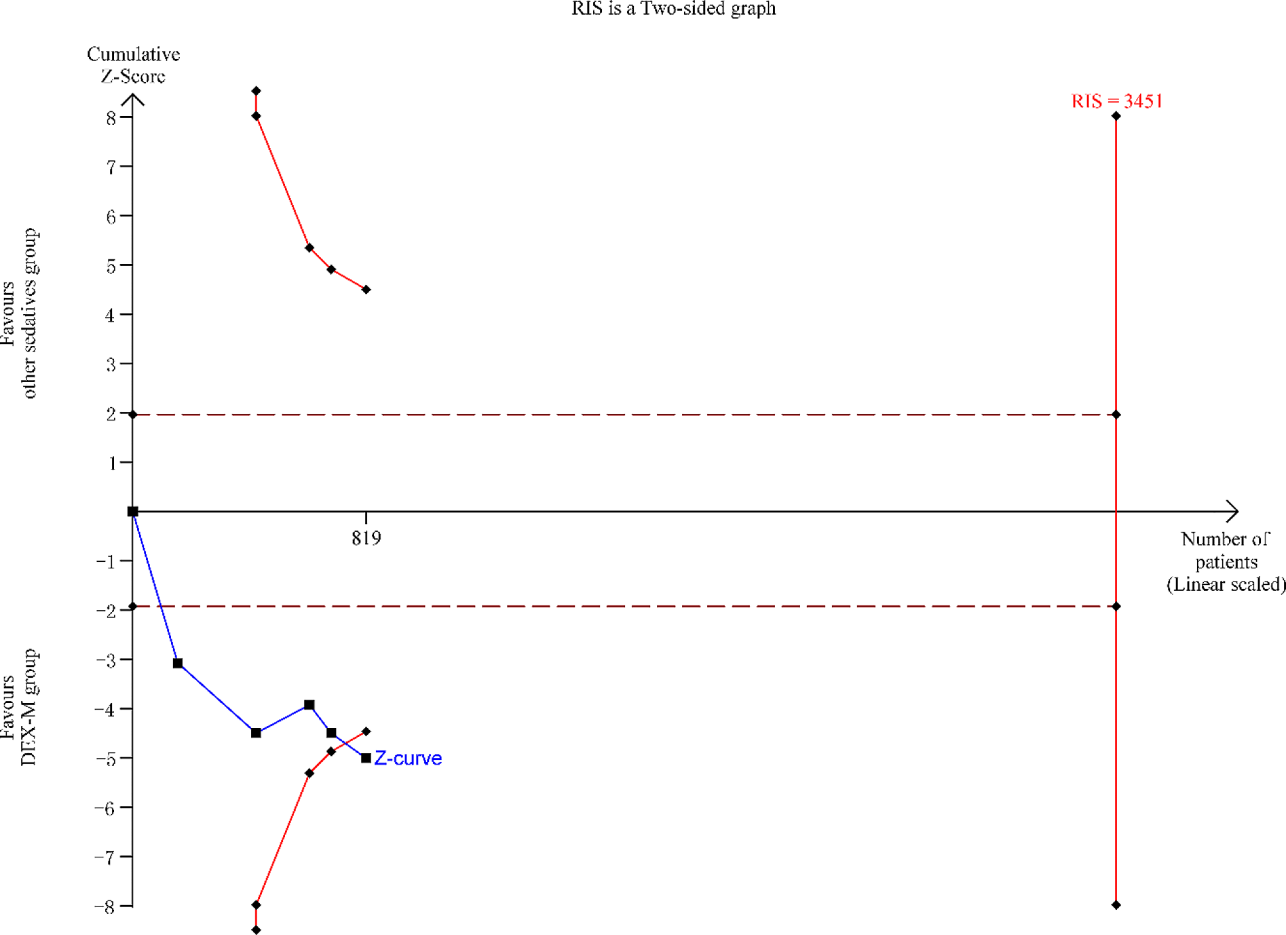
Line graph showing trial sequential analysis for the success rate of sedation in included randomized controlled trials. RIS = the required information size

The funnel plot (Fig. 6) exhibits asymmetrical distribution of study results, skewed towards the right side. This suggests a potential publication bias in the included articles, possibly indicating an overestimation of success rate of sedation in smaller studies. The results of the TSA analysis are shown in Fig. 7, the horizontal dashed line represents the traditional boundary for statistical significance. The red curve indicates the futility boundary. The cumulative z-curve represents the trial data. The results indicate that Z-curve crossed both the traditional threshold and the TSA threshold, but the cumulative information size did not reach the expected value. However, due to a certain degree of heterogeneity and publication bias among the included studies, it indicates that there is some uncertainty in the reliability of the current conclusions. Further verification and refinement of the analysis results will require more large-sample, high-quality studies in the future.

## Discussion

This meta-analysis demonstrated that, compared to the control group, a combination of DEX and midazolam resulted in a higher sedation success rate and a lower incidence of nausea and vomiting during CT, MRI, ABR, or fiberoptic bronchoscopy examinations. However, there was no significant difference in sedation recovery time or in the rates of hypotension or bradycardia between the two groups. Additionally, subgroup analysis of ASA I-II children revealed that the dexmedetomidine-midazolam group had a quicker onset time compared to other sedatives, with a P-value of 0.0001.

Midazolam, known for its pharmacological activity as a benzodiazepine, is among the most common sedatives used in the pediatric population. It is frequently utilized to induce sleep during diagnostic examinations or surgical operations [19]. This medication is effective in providing sedation, anti-anxiety, anti-convulsion, hypnosis, and muscle relaxation benefits, and is notable for its quick onset and rapid recovery [21]. However, previous studies have indicated that midazolam may lead to respiratory depression, with the risk of such depression being directly proportional to the dosage [22]. Additionally, ketamine is also very commonly used in pediatric patient due to its ability to maintain hemodynamic stability and spontaneous breathing [23], with a mild bronchodilator effect [24]. Its dissociative properties make it especially useful in procedures where the patient needs to stay still yet without complete loss of consciousness. However, the side effects of ketamine can include hallucinations, emergence reactions, and increased salivation [25]. Moreover, there is a potential for hemodynamic instability and respiratory depression, particularly at higher doses or in susceptible individuals [25]. Consequently, exploring alternative sedatives becomes crucial in order to improve the safety of sedation. In this regard, the use of alternative anesthetics such as DEX for sedation is gaining importance. Recently, there has been an increasing focus on the sedative properties of DEX in children.

DEX was approved by the Food and Drug Administration of the United States (FDA) at the end of 2008 [26] as a novel, highly efficient, and highly selective α2 adrenergic receptor agonist. The selectivity ratio for the α2-adrenoceptor to the α1-adrenoceptor is 1600:1, making it a notable sedative. DEX primarily acts on the locus coeruleus and induces natural non-rapid eye movement sleep, having less impact on breathing [27]. Nowadays, DEX has been increasingly used for procedural sedation or anesthetic premedication in children [28]. DEX can be administered by intravenous, oral, mucosal, or intramuscular routes [29]. In recent years, the intranasal route of administration for DEX has become increasingly popular due to its non-invasive nature and absence of nasal stimulation. It is rapidly absorbed owing to the rich capillary plexus in the nasal cavity, by passing the first-pass metabolism in the liver [30]. And plasma concentrations of giving intranasally or intravenously have similar pharmacokinetic properties [31]. Additionally, extra medication (with a dose range of 1–4 µg/kg, typically 1 µg/kg) may be administered in some cases of sedation failure [32]. Some researchers have found that intranasal administration of 2–3 µg/kg DEX can enable 60-82.5% of children to achieve deep sedation without significant effects on blood pressure or heart rate. In their study, among the 115 children undergoing transthoracic echocardiography examination, 100 (87%) experienced satisfactory sedation with intranasal DEX at 3 µg/kg, with only one patient requiring oxygen supplementation and all other children needing no medical intervention [33]. However, the success rate was low when intranasal DEX was used for examination or procedures with longer duration and more intense stimulation [34, 35].

Based on the results of the meta-analysis, there may be several advantages associated with combining DEX with midazolam. Firstly, the combination may be associated with more sedation success in pediatric sedation, which could be beneficial for diagnosis and procedures requiring high-intensity stimulation and lasting a long time. Secondly, DEX can induce a sedative state close to physiological sleep, while midazolam has the effect of anterograde amnesia [36, 37]. This combination might offer additional advantages for clinical diagnosis and procedures. Thirdly, the dexmedetomidine-midazolam group is not associated with an increase in adverse events, but is associated with reducing the incidence of nausea and vomiting. Finally, according to the results, this combination has a faster onset time in children classified as ASA I-II, and the recovery time is comparable to that of other sedatives included in the study.

There were several potential limitations in the present study. First, a limitation of our meta-analysis is that it may not have included the most recent research findings, indicating a need for further updates in future studies. Secondly, the studies included are randomized controlled trials, with differences in study designs and inclusion criteria. Hence, there remains a certain risk of bias among the studies. The funnel plot shows a lack of symmetry in the distribution of included studies, suggesting a potential publication bias in the articles examined and possibly an overestimation of the success rate of sedation in smaller studies. Thirdly, the number of studies included is relatively small; all are single-center clinical trials with small sample sizes. Therefore, conducting multi-center clinical trials with larger sample sizes is necessary. The meta-analysis results indicated that compared with other sedatives, the incidence of nausea and vomiting was lower in children who received DEX combined with midazolam, while the occurrence rates of bradycardia and hypotension were similar between the two groups. However, given the extremely limited sample size, more research is required to reach a definitive and reliable conclusion. Thus, future research should focus on evaluating the safety of DEX combined with midazolam in pediatric sedation, in addition to determining the optimal dosage and method of administration.

## Data Availability

The datasets generated during or analyzed during the current study are available by contacting the first authors via E-mail: 13518289033@163.com.
